# Simultaneous Open Surgical Release for Coexistent Lateral and Medial Epicondylitis in the Same Elbow: A Case Report

**DOI:** 10.7759/cureus.102073

**Published:** 2026-01-22

**Authors:** K S Arif, Vishwas Kadambila, Yunus Salim C M, Mohammed Tameez Ud Din, John Joe Jacob

**Affiliations:** 1 Orthopaedics, Kanachur Institute of Medical Sciences, Mangalore, IND

**Keywords:** elbow tendinopathy, golfer’s elbow, lateral epicondylitis, medial epicondylitis, open release, simultaneous surgery, tennis elbow

## Abstract

Lateral epicondylitis (tennis elbow) and medial epicondylitis (golfer’s elbow) are common overuse tendinopathies of the elbow, yet simultaneous clinically relevant involvement of both epicondyles in the same elbow requiring surgery is rarely described. We report the case of a 49-year-old right-hand-dominant male patient with chronic right elbow pain for three years with no antecedent trauma. He had received four corticosteroid injections around the elbow with only transient improvement. Examination revealed localized tenderness over both the lateral and medial epicondyles with pain reproduced by resisted wrist extension and flexion (Cozen’s and reverse Cozen’s tests positive). Ultrasound demonstrated focal tendinosis of the deep fibers of the common flexor origin at the medial epicondyle and a focal partial tear with tendinosis of the common extensor origin at the lateral epicondyle. After failure of prolonged conservative management, the patient underwent open debridement and release of both the common extensor and common flexor origins in a single sitting with gentle epicondylar decortication. Histopathology confirmed tendinosis with collagen and hyaline degeneration, occasional fibroblasts, and focal neovascularity, consistent with a chronic angiofibroblastic tendinopathy. The postoperative course was uneventful, and a structured rehabilitation protocol was followed. The patient reported complete relief of pain and full return to activities of daily living without recurrence. This case illustrates the presentation of concomitant, clinically significant lateral and medial epicondylitis in a single elbow in a patient who had received repeated corticosteroid injections and had prolonged symptoms. In carefully selected patients with bilateral epicondylar involvement unresponsive to non-operative care, simultaneous open debridement and release of both the common extensor and common flexor origins can provide durable pain relief and functional recovery. Routine examination of both epicondyles and critical appraisal of repeated corticosteroid injections are important when managing chronic elbow pain.

## Introduction

Lateral epicondylitis (tennis elbow) and medial epicondylitis (golfer’s elbow) are common overuse tendinopathies characterized by pain at the origins of the common extensor and common flexor-pronator tendons, respectively. Contemporary understanding recognizes these conditions as degenerative tendinopathies rather than purely inflammatory “-itis” processes, with histopathology showing disorganized collagen, hyaline degeneration, increased ground substance, and neovascularization, features originally described as angiofibroblastic tendinosis [1]. 

Lateral epicondylitis is among the most frequent causes of elbow pain in adults, with a prevalence estimated at approximately 1% to 3% in the general population and higher rates in racquet sport athletes and workers performing repetitive wrist extension and gripping [2]. It typically presents with insidious onset lateral elbow pain aggravated by resisted wrist extension and gripping, often resolving within six to 12 months in many patients, although a subset develops chronic, refractory symptoms [3]. 

Medial epicondylitis is less common, accounting for roughly 10% to 20% of epicondylitis cases, and is associated with repetitive wrist flexion, forearm pronation, and valgus stress, especially in throwing athletes and manual laborers [4]. As with lateral disease, histologic studies demonstrate a degenerative tendinosis of the flexor-pronator origin with minimal inflammatory cell infiltrate. 

Recent epidemiologic and observational work on elbow tendinopathy has highlighted that patients can show combined medial and lateral involvement and that elbow tendinopathy frequently coexists with pathology in adjacent joints [5]. Similarly, a recent platelet-rich plasma (PRP) study specifically compared outcomes of simultaneous lateral and medial epicondylitis with isolated lateral or medial disease, implying that such combined presentations are clinically relevant but form only a subset of all epicondylitis cases. Simultaneous clinically meaningful involvement of both epicondyles in the same elbow is recognized but appears relatively uncommon, forming a distinct subset of epicondylitis cases [6]. 

Although surgical management of isolated lateral or medial epicondylitis is well described, there is limited literature focusing on simultaneous open surgical release of both epicondyles in the same elbow. Case reports have described complex combined elbow pathologies, such as the triad termed “Olympic elbow” (lateral epicondylitis, cubital tunnel syndrome, and distal biceps tendon rupture), but not isolated combined epicondylitis treated with the straightforward open debridement approach used here [7]. 

We present a case of chronic, recalcitrant right elbow pain due to coexistent lateral and medial epicondylitis, both confirmed clinically, radiologically, and histopathologically, managed with single-stage open debridement and release of both the common extensor and common flexor origins, resulting in sustained symptom resolution at one-year follow-up. 

## Case presentation

A 49-year-old right-hand-dominant male presented with right elbow pain of three years’ duration. There was no history of discrete trauma. The pain was localized to both the lateral and medial aspects of the elbow, exacerbated by lifting, gripping, and activities involving wrist flexion and extension. He reported significant interference with work-related tasks and recreational activities. 

The patient had previously undergone structured non-operative treatment, including activity modification, oral non-steroidal anti-inflammatory drugs, physiotherapy focused on eccentric strengthening and stretching, and bracing. Additionally, he had received four corticosteroid injections around the elbow over this period, administered at outside facilities, with only transient or minimal relief. There was no history of inflammatory arthropathy, systemic disease, or neurologic symptoms suggestive of radiculopathy or peripheral nerve compression. 

On examination, there was localized tenderness over the lateral epicondyle at the origin of the common extensor tendon and the medial epicondyle at the common flexor-pronator origin. The Cozen’s test (resisted wrist extension with the elbow in extension) reproduced the patient’s lateral elbow pain, and the reverse Cozen’s test (resisted wrist flexion and forearm pronation) reproduced medial elbow pain. There was no instability, no effusion, and the range of motion at the elbow was full and painless at the extremes. Distal neurovascular examination was normal with no evidence of ulnar neuropathy or other neurologic deficit. 

**Figure 1:**
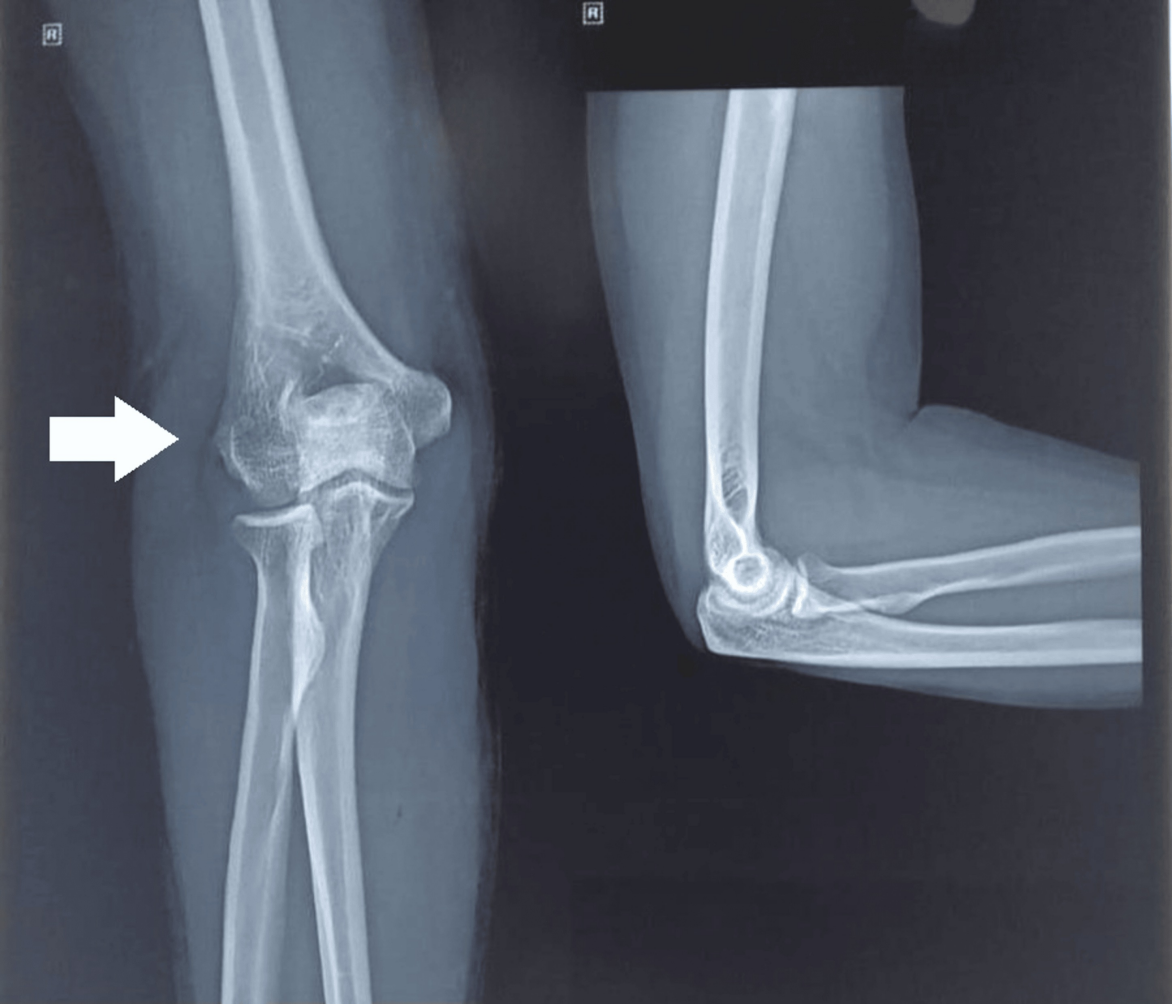
Elbow Radiograph Osteophytes, calcific tendinopathy, or arthritic changes not visualised

Plain radiographs of the right elbow (Figure 1) did not reveal osteophytes, calcific tendinopathy, or arthritic changes. 

High-resolution ultrasound demonstrated focal tendinosis of the deep common flexor origin fibers medially. At the lateral epicondyle, a focal partial tear (~4 mm sagittal, 2.4 mm axial, 2.9 mm coronal) within the common extensor origin was identified alongside tendinosis.

The clinical findings and ultrasonographic report were suggestive of coexistent medial and lateral epicondylitis involving the same elbow. Given the chronicity of symptoms, failure of extended conservative management, and significant functional limitation, operative treatment was offered. The patient consented to simultaneous open surgical management of both epicondyles. 

Operative technique 

Under regional anesthesia with tourniquet control, the patient was placed supine with the right upper limb on an arm board. The lateral epicondyle approach was as follows: A standard lateral incision was made over the lateral epicondyle. The common extensor origin was exposed, and the degenerated portion of the tendon was identified. Open debridement of the diseased extensor origin was performed, with release of the pathologic fibers while preserving as much healthy tendon as possible. Gentle “nibbling” decortication of the lateral epicondyle was carried out to create a bleeding bone bed to enhance healing. The extensor mechanism was repaired as needed, and the wound was closed in layers. 

The medial epicondyle approach was as follows: A separate medial incision was made centered over the medial epicondyle, taking care to protect the ulnar nerve. The common flexor-pronator origin was exposed, and areas of obvious tendinosis were debrided. A limited release of the degenerated fibers was performed, again combined with gentle decortication of the medial epicondyle to promote a vascular bed. The flexor-pronator origin was repaired where necessary, and the wound was closed in layers. No intraoperative complications occurred. 

Histopathology 

Representative samples from both the common extensor and common flexor origins were submitted for histopathological examination.

**Figure 2:**
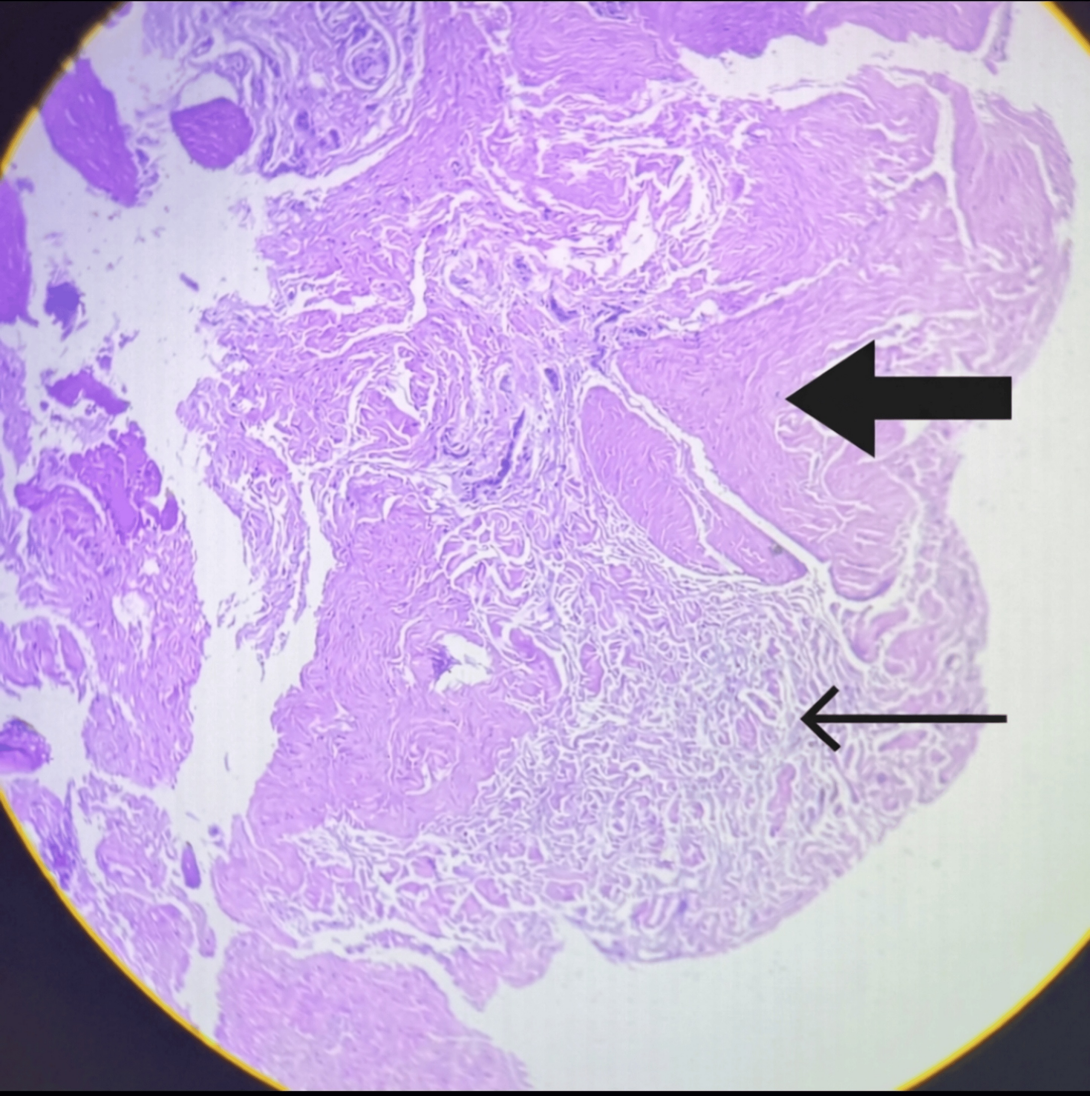
Histopatholgy Picture The thick arrow shows hyaline degeneration-homogeneous, pink, structureless with loss of normal fibrillar architecture and eosinophilic, “glassy” matrix. The thin arrow shows collagen, wavy and fibrillar, a relatively preserved area of tendon collagen.

Microscopy demonstrated collagen and hyaline degeneration, occasional fibroblasts, and focal areas showing a few small blood vessels, consistent with chronic angiofibroblastic tendinosis rather than acute inflammatory tendinitis (Figure 2).

No features of infection, crystal deposition, or inflammatory arthropathy were seen. 

Postoperative course and follow-up 

The postoperative period was uneventful. The elbow was temporarily immobilized in a posterior splint for pain control, followed by early gentle range-of-motion exercises as per standard rehabilitation protocol. Progressive strengthening exercises were initiated once wound healing was satisfactory and pain permitted. The patient was reviewed at two weeks, six weeks, three months, six months, and one year postoperatively. At the final follow-up at one year, he reported complete resolution of elbow pain in day-to-day activities, return to full activities of daily living, and no recurrence of symptoms on either the medial or lateral side. There were no wound-related complications, no neurologic deficit, and no subjective or objective instability.

## Discussion

This case highlights several important aspects of elbow tendinopathy: the degenerative nature of both lateral and medial epicondylitis, the possibility of simultaneous involvement of both epicondyles in a single elbow, the potential negative implications of repeated corticosteroid injections, and the role of single-stage open debridement and release of both sides in carefully selected refractory cases. 

Pathophysiology and histopathology 

Both lateral and medial epicondylitis are now widely regarded as chronic tendinopathies characterized by degenerative changes at the tendon origin rather than classical inflammatory “tendinitis.” The original description of angiofibroblastic tendinosis by Kraushaar and Nirschl remains a landmark in understanding tendon pathology, with hallmark features of disorganized collagen, mucoid change, fibroblast proliferation, and neovascularization [1]. 

Recent high-quality reviews have reinforced that lateral epicondylitis is a degenerative condition of the extensor carpi radialis brevis origin, often self-limiting but frequently recurrent, with pain mediated by nociceptive and possibly neurogenic mechanisms [2,3]. Similarly, medial epicondylitis has been described as a degenerative tendinosis of the flexor-pronator origin, most often involving the pronator teres and flexor carpi radialis, again with histologic findings of collagen disorganization, fibroblast proliferation, and neovascularity but limited inflammatory infiltrate [4]. In our case, the histopathology from both origins demonstrated features classic for chronic tendinosis, collagen/hyaline degeneration, fibroblasts, and focal neovascularization, thereby aligning with the angiofibroblastic tendinosis model described by Kraushaar and Nirschl [1] rather than an acute inflammatory process.

Epidemiology and co-occurrence of lateral and medial epicondylitis 

Lateral epicondylitis is one of the most common upper limb tendinopathies, affecting approximately 1% to 3% of adults and peaking in middle age [2,3]. It has been associated with repetitive wrist extension, gripping, and forceful forearm use in both athletes and workers and is a frequent cause of work disability [2,3]. Medial epicondylitis is less frequent but occurs particularly in individuals performing repetitive wrist flexion, pronation, and valgus-loading activities, such as throwers and certain manual laborers [4]. 

Recent work has emphasized that elbow tendinopathy is often multifocal, involving both medial and lateral compartments or coexisting with pathology in the shoulder or wrist. A multicenter observational study of lateral and medial elbow tendinopathy showed that many patients had previous or concomitant injuries in adjacent joints and highlighted the complex, sometimes widespread nature of upper limb tendinopathy [5]. 

Park et al. evaluated PRP treatment outcomes in patients with simultaneous medial and lateral epicondylitis compared with isolated lateral or medial disease, again confirming that combined involvement can occur, although it represents only a proportion of epicondylitis cases. In that study, simultaneous involvement could be treated effectively with PRP injections, though functional improvement appeared somewhat attenuated compared with single-site disease [6]. 

However, despite recognition that medial and lateral epicondylitis can occur together, specific reports of pure combined medial and lateral epicondylitis in the same elbow treated with simultaneous open debridement of both epicondylar origins are sparse. To the best of our knowledge, after a focused search of recent literature, such cases are rarely documented, particularly with detailed histopathology and structured one-year follow-up. 

Non-operative management and indications for surgery 

For both lateral and medial epicondylitis, initial management is typically non-operative and includes activity modification, non-steroidal anti-inflammatory drugs, physiotherapy (especially eccentric strengthening), bracing, and various injection therapies (corticosteroid, PRP, autologous blood, or other orthobiologics) [2,5]. A recent review in the New England Journal of Medicine emphasizes that most cases of lateral epicondylitis resolve within six to 12 months and that no single non-operative modality has shown a clearly superior long-term benefit [2]. Comprehensive reviews of lateral and medial epicondylitis similarly advocate a stepwise non-operative approach, reserving surgery for patients with persistent pain and functional impairment despite prolonged conservative care [3,4]. 

Our patient had three years of symptoms, had undertaken standard non-operative measures, and had received four corticosteroid injections with inadequate, durable relief. Repeated corticosteroid injections have been associated with transient pain relief but can potentially impair tendon healing and are linked to poorer long-term outcomes in tendinopathy, including an increased risk of structural compromise in some tendon locations [2]. Although a direct causal relationship cannot be established in a single case, it is reasonable to consider that multiple corticosteroid injections may have contributed to the progression of tendon degeneration in this patient, reinforcing the need for judicious use of steroids in chronic elbow tendinopathy. 

Surgical management of lateral epicondylitis 

When conservative treatment fails, surgical options for lateral epicondylitis include open, percutaneous, and arthroscopic debridement and/or repair of the common extensor origin. Systematic reviews have shown that all three approaches generally produce good to excellent outcomes, with high rates of pain relief and return to work or sport, but no clearly superior technique [7,8]. Burn et al. reported favorable outcomes with open, arthroscopic, and percutaneous procedures, with improvements in pain and function across all modalities [7]. Li et al. conducted a systematic review and meta-analysis comparing open and arthroscopic techniques and found no significant difference in clinical outcomes, with both approaches achieving reliable symptom relief [8]. 

More recently, Mirvish and Fowler reported on the surgical management of lateral epicondylitis using a standardized technique, demonstrating high satisfaction and functional improvement with low complication rates at follow-up [9]. These studies support the role of debridement of degenerative tissue with or without repair, often combined with limited decortication of the epicondyle to stimulate a vascular bed, in carefully selected patients with recalcitrant lateral epicondylitis. In the present case, the open lateral approach allowed direct visualization and debridement of the degenerated common extensor origin and controlled epicondylar decortication, consistent with the principles described in contemporary literature [7-9]. 

Surgical management of medial epicondylitis 

Surgical treatment of medial epicondylitis is less extensively reported than that of lateral epicondylitis but generally follows similar principles: debridement of diseased tendon, partial release or repair of the flexor-pronator origin, and protection of the ulnar nerve and medial collateral ligament. DeLuca et al. and other recent reviews summarize current diagnostic and management approaches, emphasizing that surgery is reserved for patients with severe, refractory symptoms and imaging evidence of tendon degeneration or tearing [4].

A systematic review by Arevalo et al. examined surgical techniques and outcomes for medial epicondylitis and identified three main approaches: open, arthroscopic, and percutaneous. Regardless of technique, patients typically demonstrated substantial improvements in patient-reported outcome measures and return to work or sport, with relatively low complication rates [10]. More recently, Barakat et al. performed a systematic review of surgical techniques for medial epicondylitis, highlighting overall good postoperative outcomes with low complication rates across open, arthroscopic, and percutaneous approaches, and suggesting that preoperative ulnar neuritis and prior injections did not consistently worsen surgical results [11]. Our medial approach, open debridement of degenerative tissues, limited release of the common flexor-pronator origin, and gentle epicondylar decortication while protecting the ulnar nerve, is in keeping with techniques described in these contemporary series [10,11]. 

Combined pathology and rationale for simultaneous surgery 

Recent literature confirms that simultaneous medial and lateral epicondylitis can occur clinically and that both sites may respond to biologic treatments such as PRP when addressed together. Park et al. reported that concurrent medial and lateral epicondylitis treated with PRP showed meaningful pain reduction, although functional gains were somewhat less than in isolated lesions [6]. Vinolo-Gil et al. further demonstrated that elbow tendinopathy frequently coexists with pathology in adjacent joints, underscoring the broader, sometimes systemic or kinetic-chain nature of upper limb tendinopathy [5]. Case-based descriptions such as the “Olympic elbow” triad illustrate that chronic lateral epicondylitis may coexist with other elbow pathologies and, after failure of non-operative treatments including corticosteroid injections, can progress to more complex injury patterns amenable to surgical correction [12]. 

In our patient, both epicondyles were clearly symptomatic, with concordant clinical tests and ultrasound evidence of focal tendinosis and partial tearing. Non-operative therapy, including multiple corticosteroid injections, had failed over three years. In this setting, addressing only one epicondyle would likely have left residual pain due to the other lesion. We, therefore, elected to perform a single-stage open debridement and release of both the common extensor and common flexor-pronator origins, with limited epicondylar decortication on each side. 

The excellent clinical outcome at one-year follow-up in this case suggests the potential for simultaneous open surgery to be a safe and effective option for combined medial and lateral epicondylitis in carefully selected patients, avoiding the need for staged procedures when both lesions are clearly symptomatic and confirmed by imaging, non-operative treatment has been exhausted, and there is no significant concomitant instability or neurologic compromise requiring a different surgical strategy. 

Learning points 

Always examine both epicondyles in patients presenting with chronic elbow pain. Coexistent medial and lateral epicondylitis may be under-recognized and can explain persistent symptoms when only one side is initially suspected. Repeated corticosteroid injections should be used cautiously in chronic tendinopathy. Although they may provide short-term relief, they have not demonstrated durable benefit and may be associated with tendon weakening and progression of structural pathology [2,3,9]. In appropriately selected patients with combined epicondylar tendinopathy refractory to comprehensive conservative care, single-stage open debridement and release of both the common extensor and common flexor-pronator origins can be a rational strategy, avoiding staged operations and potentially facilitating a unified rehabilitation program. 

## Conclusions

This case demonstrates that clinically significant lateral and medial epicondylitis can coexist in the same elbow and present as chronic refractory pain after repeated corticosteroid injections and prolonged symptoms. In such a scenario, simultaneous open debridement and release of both epicondylar origins, combined with limited epicondylar decortication and structured rehabilitation, can provide durable pain relief and restoration of function. Recognition of combined pathology, cautious use of corticosteroid injections, and timely consideration of surgical intervention for recalcitrant cases may prevent prolonged disability and improve functional outcomes in patients with complex elbow tendinopathy.

## References

[1] Kraushaar BS, Nirschl RP (1999). Tendinosis of the elbow (tennis elbow). Clinical features and findings of histological, immunohistochemical, and electron microscopy studies. J Bone Joint Surg Am.

[2] Wolf JM (2023). Lateral epicondylitis. N Engl J Med.

[3] Konarski W, Poboży T (2023). A clinical overview of the natural course and management of lateral epicondylitis. Orthopedics.

[4] DeLuca MK, Cage E, Stokey PJ, Ebraheim NA (2023). Medial epicondylitis: current diagnosis and treatment options. J Orthop Rep.

[5] Vinolo-Gil MJ, García-Campanario I, Estebanez-Pérez MJ, Rodríguez-Huguet M, Linares-Gago M, Martin-Vega FJ (2024). Lateral and medial elbow tendinopathy and previous injuries to adjacent joints: a multicenter observational study. Healthcare (Basel).

[6] Park HS, Jo SM, Dasari S, Moon YL (2023). Comparison of clinical outcomes of platelet-rich plasma for epicondylitis, elbow: simultaneous lateral and medial versus lateral versus medial. Orthop Surg.

[7] Burn MB, Mitchell RJ, Liberman SR, Lintner DM, Harris JD, McCulloch PC (2018). Open, arthroscopic, and percutaneous surgical treatment of lateral epicondylitis: a systematic review. Hand (N Y).

[8] Li Y, Guo S, Li S, Yang G, Lu Y (2023). Is there any difference in clinical outcome between open and arthroscopic treatment for tennis elbow? A systematic review and meta-analysis. Orthop Surg.

[9] Mirvish AB, Fowler JR (2024). Surgical management of lateral epicondylitis: a retrospective review of technique success. Hand (N Y).

[10] Arevalo A, Rao S, Willier DP 3rd (2023). Surgical techniques and clinical outcomes for medial epicondylitis: a systematic review. Am J Sports Med.

[11] Barakat A, Jha G, Raval P, Abourisha E, Divall P, Singh HP, Pandey R (2025). Systematic review of surgical techniques for medial epicondylitis: evaluating the impact of preoperative injections and concomitant ulnar neuritis on postoperative outcomes. Ann R Coll Surg Engl.

[12] Gencarelli P Jr, Mittal R, Yi R, Lee JM Jr (2023). Olympic elbow comprising lateral epicondylitis, cubital tunnel syndrome, and distal biceps tendon rupture. Cureus.

